# Multi-Frame Star Image Denoising Algorithm Based on Deep Reinforcement Learning and Mixed Poisson–Gaussian Likelihood

**DOI:** 10.3390/s20215983

**Published:** 2020-10-22

**Authors:** Ming Xie, Zhenduo Zhang, Wenbo Zheng, Ying Li, Kai Cao

**Affiliations:** Navigation College, Dalian Maritime University, Dalian 116026, China; mingxie@dlmu.edu.cn (M.X.); zhangzhenduo@dlmu.edu.cn (Z.Z.); zwb123456789@dlmu.edu.cn (W.Z.); caokai@dlmu.edu.cn (K.C.)

**Keywords:** star image, image denoising, reinforcement learning, maximum likelihood estimation, mixed Poisson–Gaussian likelihood

## Abstract

Mixed Poisson–Gaussian noise exists in the star images and is difficult to be effectively suppressed via maximum likelihood estimation (MLE) method due to its complicated likelihood function. In this article, the MLE method is incorporated with a state-of-the-art machine learning algorithm in order to achieve accurate restoration results. By applying the mixed Poisson–Gaussian likelihood function as the reward function of a reinforcement learning algorithm, an agent is able to form the restored image that achieves the maximum value of the complex likelihood function through the Markov Decision Process (MDP). In order to provide the appropriate parameter settings of the denoising model, the key hyperparameters of the model and their influences on denoising results are tested through simulated experiments. The model is then compared with two existing star image denoising methods so as to verify its performance. The experiment results indicate that this algorithm based on reinforcement learning is able to suppress the mixed Poisson–Gaussian noise in the star image more accurately than the traditional MLE method, as well as the method based on the deep convolutional neural network (DCNN).

## 1. Introduction

A star image is obtained from star sensor, which is a high-accuracy attitude determination instrument. The three-axis attitude and spatial position of the star sensor can be calculated based on the reference of stars recognized in the digitized star image. Being able to accurately collect and process a star image at all times, however, is one of the challenges in the application of a star sensor due to the brightness of the sky’s background and the complicated mixture of noise [1]. These conditions lead to a low Signal-to-Noise Ratio (SNR) in the star image obtained in the daytime, and thus have negative influences on the calculation of attitude and position. Similarly, it is an important basis for the star sensor to work efficiently that noise is effectively suppressed and the star targets are accurately recognized in the star image. Researchers have proposed various types of algorithms to suppress the effects of salt-and-pepper noise, strip noise, speckle noise, and defective pixels [1,2,3,4,5]. Nevertheless, mixed Poisson–Gaussian noise remains after these denoising methods are applied and affects the extraction of the true value of star points [6].

The traditional method suppresses the mixed Poisson–Gaussian noise simply by taking the arithmetic mean of multiple frames of star images. However, the restoration through such methods is not likely to be accurate without a large number of star images that are taken in a short period or by a stational star sensor [7]. Considering the movement in the carrier of the star sensor, such conditions could sometimes be unrealistic. Maximum likelihood estimation (MLE) is one of widely used methods that can restore a star image with limited number of frames. In the MLE method, the distribution model of the noise is constructed as a likelihood function, and the image that is most likely to produce the given observations is termed as the restored image. However, mixed Poisson–Gaussian noise is still difficult to be suppress through the MLE method due to its complicated likelihood function. This will be discussed in detail in the next section.

Towards the goal of high-accuracy star image restoration, this study integrated deep reinforcement learning techniques with the MLE method and developed an iterative denoising algorithm that can accurately suppress mixed Poisson–Gaussian noise. Generally, an agent proposes “restored images”, which are evaluated through a modified likelihood function. Borrowing the idea of the MLE method, the mission of the agent is to find the image that returns the highest probability according to the modified likelihood function. Based on the gradient descent algorithm in the deep reinforcement learning method, the agent efficiently learns to propose a more appropriate “restored image” until it gets close enough to the ground truth. Thus, the aim and contribution of this study is to build a fully automated denoising algorithm that can accurately suppress the mixed Poisson–Gaussian noise in a star image.

## 2. Related Works

### 2.1. The Application of MLE in Star Image Denoising

The MLE method regards the image denoising problem as the optimization process of the likelihood function. Moreover, other regularization terms can be added to the likelihood function as the constraints on the estimator and form a denoising algorithm together with the likelihood function. Based on the MLE method and the image blur model with Gaussian noise, Katsaggelos proposed an image restoration algorithm using blur coefficients identification and expectation-maximization (EM) [8]. Llacer and Nunez applied the MLE method to restore the astronomic images obtained from the Hubble Space Telescope and proposed an iterative MLE and Bayesian algorithm [9]. Similarly, Synder discussed the convolution of astronomic image obtained from Charge Coupled Device (CCD) camera and suggested that the restoration of such astronomic image could potentially be achieved via EM method [10]. Benvenuto further explored the image denoising problem specifically for star images. They gave an approximate model of the flux of photons and verified the existence of its solution [11]. Li et al. constructed the likelihood function for multi-frame Adaptive Optics (AO) image according to the Poisson distribution model, based on which they proposed an AO image denoising method that achieved accurate results [12]. However, this algorithm is not able to restore the image with mixed Poisson–Gaussian noise, which has a more complicated distribution model. Zhang et al. approximated the mixed Poisson–Gaussian noise model using generalized Anscombe transformation approximation Fourier ptychographic (GATFP), and then solved the transformed likelihood function with the MLE method [6]. Although the GATFP method is able to optimize the complicated distribution model of mixed Poisson–Gaussian noise, it cannot avoid the error introduced during the approximation.

### 2.2. Reinforcement Learning and Its Application in Image Processing

Derived from behavioral psychology, reinforcement learning is a classical topic in the studies of artificial intelligence (AI) [13,14]. Various algorithms have been developed for reinforcement learning, but they share a major process in common: an agent learns through the interactions with certain environment, and gradually finds an optimal solution or approach. The Markov Decision Process (MDP) is a commonly used method of reinforcement learning [15]. In MDP, an agent takes limited choices of actions to update the state of the environment, then receives encouragement or punishment according to a reward function that evaluates the chosen action, as well as how it changed the state [16]. As it can be inferred from the learning process, MDP is usually accomplished through an iterative algorithm.

Due to the development of AI technology in the last decade, the machine learning algorithm has been applied in many subjects, including object detection [17,18,19], data mining [20,21], image processing [22,23], etc. Typically, the convolutional neural network (CNN) has been successfully applied in various image processing tasks, including deblurring [24,25,26], denoising [27,28,29], JPEG artifacts reduction [30,31,32], and super-resolution [33,34,35,36,37,38,39,40], owing to its ability to efficiently handle imagery information. Incorporated with the deep learning algorithm, traditional reinforcement learning also rapidly developed [41,42] and showed its potential of application in image processing [43,44]. Yu et al., designed a toolbox for image processing, and then developed an image restoration algorithm based on reinforcement learning using the tools within the toolbox [44]. Despite its various applications in image processing, the machine learning method is not widely utilized in star image restoration. This is partly because the star image restoration problem is different from the general image restoration problem: the goal is to restore the value of a star point as close to the true value as possible, rather than make the image visually fine. A deep convolutional neural network (DCNN) model proposed by Liu et al. is one of the limited examples of applying deep learning method in star image denoising [45]. Liu et al. trained a supervised DCNN model with simulated star image and achieved better restoration than traditional method.

### 2.3. Mixed Poisson–Gaussian Noise in Star Image

Gaussian noise in the star image can be introduced by many aspects including the noise of circuit components, the temperature change in image sensor, etc. The intrinsic noise introduced during the photon counting process of the photosensitive component, however, depends on the signal value, thus follows Poisson distribution. Because the measurements from all pixels are independent, for a star image with *M* × *M* pixels and *N* observations, the likelihood function that describe the possibility of receiving the known *N* observations is given as Equation (1) [46]:

(1) $$p(z_{q}|b_{q})=\prod_{q=1}^{N}\prod_{i=1}^{M^{2}}(\sum_{j=1}^{+\infty }\frac{e^{-{\left[b_{q}\right]}_{i}}{\left[b_{q}\right]}_{i}^{j}}{j!}\frac{e^{-\frac{1}{\sigma^{2}}{\left({\left[z_{q}\right]}_{i}-j\right)}^{2}}}{\sqrt{2\pi \sigma^{2}}})$$

where *z_q_* is the noise image, *b_q_* is the ground truth, *σ* is the variance of Gaussian distribution, subscript *i* is an index of the number of pixels, *q* is an index of the number of observations, and *j* is an index of the possible observation of the gray value. As discussed above, MLE method is intended to find a restore image *b^′^_q_* that returns the highest value of the likelihood function.

Taking the negative logarithm of the likelihood function makes it easier to solve this problem. Thus, the optimized likelihood function is given as:

(2) $$\min f(z_{q}|b_{q}^{\prime })=-\log(\prod_{q=1}^{N}(p(z_{q}|b_{q}^{\prime }))=-\sum_{q=1}^{N}\sum_{i=1}^{M^{2}}\log(\sum_{j=1}^{+\infty }\frac{e^{-{\left[b_{q}^{\prime }\right]}_{i}}{\left[b_{q}^{\prime }\right]}_{i}^{j}}{j!}\frac{e^{-\frac{1}{\sigma^{2}}{\left({\left[z_{q}\right]}_{i}-j\right)}^{2}}}{\sqrt{2\pi \sigma^{2}}})$$

Nevertheless, it is still extremely difficult to directly solve this likelihood function. Marnissi et al., further optimized the likelihood function using generalized Anscombe transformation (GAT) approximation, and form the likelihood function as Equation (3) [47]:

(3) $$p(z_{q}|b_{q})=\prod_{i=1}^{M^{2}}\frac{1}{\sqrt{2\pi }}\exp(-\frac{1}{2}{\left(z_{q}-2\sqrt{{\left[b_{q}\right]}_{i}+\frac{3}{8}+\sigma^{2}}\right)}^{2})$$

As indicated in Equation (3), GAT method simplifies the mixed Poisson–Gaussian likelihood as a Gaussian-like distribution. Although it is able to provide an approximate solution to the likelihood function, the tails of variance stabilized coefficients distribution are still empirically longer than normality [48] (which means the variance of the transformed noise still depends on signal intensity). In this study, the mixed Poisson–Gaussian likelihood function is directly introduced in the reinforcement learning algorithm without approximation. Therefore, it is expected that the likelihood function given by Equation (2) can be solved more accurately, and the mixed Poisson–Gaussian noise in the star image can be suppressed more effectively.

## 3. Methodology

### 3.1. Dataset

In this study, simulated star images are produced as the dataset for training and testing the model. Simulated star image has been successfully utilized to train machine learning model owing to its ability of generating a variety of star images under different conditions at very low cost [45]. Xu et al., studied the stellar radiation model and proposed a star image simulation method based on calibration coefficient, which relates to stellar magnitude *m_v_* and color temperature *T* [49]. Generally, the total energy of photoelectrons generated by an observed star can be calculated by Planck’s black body radiation law as:

(4) $$E_{s}(\lambda ,T)=\frac{2\pi hc^{2}}{\lambda^{5}\left[\exp\left(hc/\lambda k_{B}T\right)-1\right]}$$

where *λ* is wavelength, *k_B_* is Boltzmann constant, *h* is Planck constant, *E_s_* is the stellar radiation energy per unit area, per unit time, and per incremental wavelength (W/m^3^).

**Figure 1:**
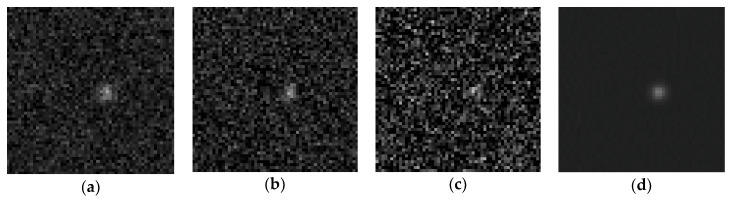
Simulated star images with: (**a**) mild noise, (**b**) moderate noise, (**c**) severe noise, (**d**) ground truth.

The energy of star point is then dispersed into several pixels based on point spread function (PSF) of the optical system. After that, the star points are integrated with the background simulated based on MODTRAN software to generate the simulated star image. Finally, mixed Poisson–Gaussian noise is added to the image. This simulation method is adopted in this study to produce the training dataset. More specifically, three levels of noise images are produced in the dataset as mild noise, moderate noise, and severe noise, in which the variances of Gaussian noise are 5, 10, and 20, respectively. The noise in each image is randomly generated and independent from each other in order to avoid having the same noisy instantiation among the different images in the dataset. The simulated image of ground truth and three sets of star images with different amount of noise is shown as Figure 1.

Two thousand different simulated star images are produced as the images of ground truth. For each star image of the ground truth, 10 noise images are produced for each type of noise. Thus, there are 6000 sets of noise images that contain three levels of noise and are produced based on 2000 different images of ground truth. Each set includes 10 frames of noise image, thus the training and testing for certain image with each ground truth image that contains each type of noise is carried out based on 10 frames of noise images. The image sets are randomly shuffled for model training. Seventy percent of the images are used for training, 10% of the images are used for validation, and 20% of the images are used for testing. The star images with different types of noise are distributed in the training, validation, and testing dataset as evenly as possible. All of the tests on hyperparameters are carried out separately with the testing images of these three types of noise.

### 3.2. Reinforcement Learning Algorithm

**Figure 2:**
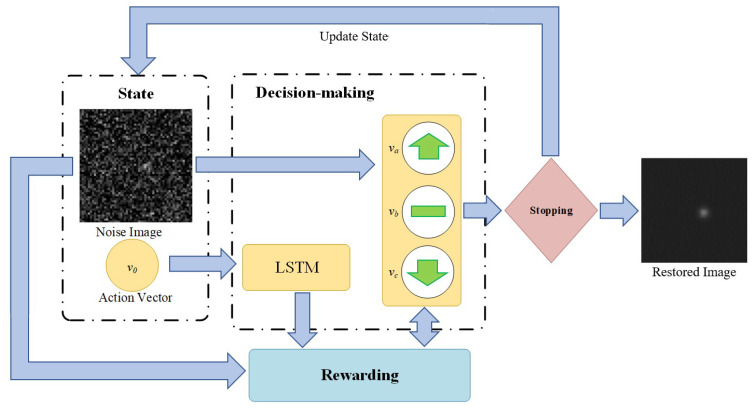
Flowchart of the star image denoising algorithm based on reinforcement learning.

Different from existing star image denoising methods (such as GAT) that try to approximately solve the likelihood function given by Equation (2), we intend to solve the denoising problem through MDP, which include an environment part (state), a decision-making part (agent), a rewarding part, and a stopping part (Figure 2). The decision-making part receives the noise image and treats it as the initial state, makes a sequence of actions on the image and produces a processed image. The rewarding part mainly consists of a reward function that returns a higher value when the processed image is closer to the ground truth. In the case of the star image denoising in this study, the reward function is directly derived from the likelihood function that is given by Equation (2). The stopping part evaluates the processed image and determines whether it is close enough to the ground truth. If the processed image does not satisfy the stopping part, it will be sent back to the decision-making part as an updated state. Otherwise, the stopping part will end the iterations and return the processed image as the final restored image. The detail settings of these parts are discussed as follows.

#### 3.2.1. State and Initial State

The state is referred as a set of input information for the decision-making part. In this study, the state consists of two sections: (1) the current input image, on which the selected actions will be directly applied; (2) the historical action vector, which is the action selected in the previous iteration. In the iterative image denoising model proposed by Yu et al., a past historical action vector is also added as a reference to the action selection at current iteration [44]. They suggest that the restoration results are improved compared with using current input image only.

The input image of the initial state is set as the arithmetic mean of the multi-frame star images. Because the average of multi-frame star images is usually not too far away from the ground truth (as discussed in the introduction, this is the traditional way to restore star image), using it as the initial input image reduces the numbers of iterations (NOI), and also avoids the local optimum. The action vector of the initial state is set as a zero vector, since there is no action taken before the initial state.

#### 3.2.2. Decision-Making Part

The decision-making part receives the input information and performs predefined choices of actions on the image. There are two modules in the decision-making part corresponding to the two sections of input state. The first module processes the input image. The star images are 8-bit or 16-bit grayscale images, of which the gray value is determined by the number of photons counted by the star sensor. Therefore, the decisions for any given pixels can be simply defined as: (1) increasing gray value by *w*; (2) decreasing gray value by *w*; (3) stay the same. The parameter *w* is the searching radius, which is (similar as the learning rate in deep neural network) a hyperparameter that can be adjusted based on the property of image. For the 8-bit grayscale images with mild noise, a searching radius can be directly set as 1, since the initial input image is usually not too far away from the ground truth. For the 16-bit gray-scale images or images with severe noise, searching radius could be set as a variable that is larger in the first few iterations, and then gradually decreases to 1 as the iterations go on. Increasing the initial searching radius (ISR) can reduce NOI when the amount of noise is relatively large, but it has to decrease to 1 in order to produce accurate restored image.

The second module analyzes the historical action vectors based on long short-term memory (LSTM) [50]. LSTM is a special type of recurrent neural network (RNN), of which the output of current state partly depends on the previous input. Compared with regular RNN, LSTM includes an additional hidden state that decides whether the previous input should be “remembered” or “forgotten” based on a gating function. By abandoning unnecessary inputs, LSTM overcomes the gradient vanishing/explosion problem in regular RNN, and achieves better performance in processing long sequence data [51]. LSTM is introduced in the model because the iterative actions performed by the decision-making part form a sequence dataset, which can be effectively processed by LSTM. By storing historical images and corresponding actions, LSTM provides additional information and references for current action selection, and enable the model to learn from previous actions.

#### 3.2.3. Rewarding Part

Theoretically, any functions that fulfill the requirement that returns a higher value as it is getting closer to the ground truth could potentially be a reward function. However, the choice of reward function significantly influences the performance of the restoration.

In the traditional reinforcement learning algorithm for image denoising, reward function is usually derived from Peak Signal-to-Noise Ratio (PSNR) or Structural Similarity (SSIM) [44]. In this study, the likelihood function of mixed Poisson–Gaussian noise, which was approximately solved in the previous studies [5,47,48], is introduced as the reward function without approximation. Specifically, the reward function is showing as follows:

(5) $$\max f(z_{q}|b_{q}^{\prime })=\log(\prod_{q=1}^{N}(p(z_{q}|b_{q}^{\prime }))=\sum_{q=1}^{N}\sum_{i=1}^{M^{2}}\log(\sum_{j=1}^{+\infty }\frac{e^{-{\left[b_{q}^{\prime }\right]}_{i}}{\left[b_{q}^{\prime }\right]}_{i}^{j}}{j!}\frac{e^{-\frac{1}{\sigma^{2}}{\left({\left[z_{q}\right]}_{i}-j\right)}^{2}}}{\sqrt{2\pi \sigma^{2}}})$$

There are two advantages of applying this likelihood function as reward function: (1) it returns highest value at the ground truth, which fulfills the requirement of reward function for reinforcement learning; (2) by directly applying the likelihood function as the reward function rather than making an approximate solution (like GAT method), it is expected to achieve more accurate restoration on the star image.

In practice, the variance of the Gaussian noise is unknown, but is required to calculate the rewards. This term needs to be calculated from the variance of the marginal pixels that are far away from stars. Because the signal for the background is expected to be close to 0 and Poisson noise is related to signal intensity, ideally the Poisson noise in the marginal pixels are ignorable, and the variance in the marginal pixels are mostly contributed by that of the Gaussian noise.

It should also be noted that there is an infinite term in the function. This term refers as the probability of the showing certain gray value at a given pixel. Therefore, it usually returns high value when the gray value is close to the ground truth, and then decreases to almost 0 when the difference is too large (it is less likely that the observed value has too much difference from the ground truth). Therefore, in our algorithm, this term is not taken into account when it is smaller than 10^−8^—the probability less than that has little influence on the reward function and is ignorable. Moreover, in practice, the differences between the possibilities calculated from Equation (5) could be very small (especially when the variance of the noise is relatively large and the curve of the likelihood function is “flat”), which means the differences between the rewards of actions are not obvious. As a result, the decision-making part may fail to determine the appropriate action due to the vague rewarding. In order to solve this problem, the values returned from the likelihood function may need to be normalized to a larger scale, so as to magnify the differences between the rewards of different states and actions.

#### 3.2.4. Stopping Part

An automatic stopping part is designed in the algorithm in order to determine whether the processed image is close enough to the ground truth, and if yes, automatically stop the iterations. According to the decision-making part and reward function described in the previous sections, it can be easily inferred that when the processed image reaches the ground truth, the decision-making part would choose to stay at the same gray value, because either increasing or decreasing the gray value would return negative rewards. Thus, stopping index (SI), defined as the numbers of times that the decision-making part has continuously chosen to stay at the same gray value, is designed as the main algorithm of the stopping part. SI is also a hyperparameter that should be adjusted based on property of the star images. For the reason that the decision-making part could possibly choose random actions, increasing SI in some extent can improve the accuracy of the restoration results, but at the same time, increase number of iterations (NOI). For the same reason, the maximum number of iterations (MNI) may also need to be defined, so as to avoid infinite loop. The detail test on SI and MNI will be discussed in the next section.

### 3.3. Implementation Details

The algorithm in this study is built using Tensorflow backend [52] and in Python 3.6 environment. Deep Q-learning [41] is adopted in this study for the training process. The *ε*-greedy is set relatively high as 0.95, because the rewarding function is continuous and has only single peak, which means taking the action with higher rewarding is a better choice than taking a random action for the most of the cases. Adam [53] optimizer is applied in the model and the batch size is set at 32. The hyperbolic tangent function is applied as the gating function of LSTM.

In order to find the favorable choice of hyperparameters, two sets of experiments were conducted to test the hyperparameters introduced above. For the tests on SI, the searching radius was set as a constant of 1, and MNI was set as a constant of 500. Six experiments were conducted for SIs, varying from 2 to 7 with an increment of 1. For the tests on the ISR, the SI is set as a constant of 3, and the MNI was set as a constant of 500. Four experiments were conducted for the test on ISR. In one of the experiments, the searching radius is set as a constant of 1. While in the other three experiments, the ISR is set at 5, 10, and 20, respectively, and then decreased at a factor of 0.5 (rounding up) every 10 iterations. The working flow and specific implementations for the algorithm proposed in this study is summarized in Algorithm 1.
**Algorithm 1.** Steps for the proposed denoising algorithm
**Step 1**: Create initial input image as the arithmetic mean of *N* frames of noise image; define initial action vector *v*_0_ as a zero vector; initialize stopping counter *c_s_* = 0; setup hyperparameter: *SI*, *MNI*, *ISR** Initialize operation**Step 2**: Calculate the variance of Gaussian noise using the boundary pixels
**Step 3**: Define action table (*a*_1_ = + *w*, *a*_2_ = − *w*, *a*_3_ = *no action*, initialize *w* equal to *ISR*); initialize action-value function *Q* with random weight *θ** Setup deep Q-learning**Step 4**: **for** each of the *M* × *M* pixels: initialize sequence of state {*b_i_*}* Start iterationIterate through *i* = 1, 2, …, *MNI*:
Process *v_i-1_* through LSTM and obtain *v^’^_i-1_*
With probability *ε* select *v_i_* = argmax *Q*(*b_i-1_*; *v^’^_i-1_*; *θ*)
Otherwise select a random action *v_i_* from action table
Execute action *v_i_*, update environment *b_i_ = b_i-1_* + *v_i_*, and observed reward *r_i_*
Update action-value function *Q* with the observed (*b_i_*; *v_i_*; *r_i_*)
After every 10 iterations *w* ← rounding up (*w*/2)
**if***a_3_* is chosen in this loop **then**: *c_s_* ← *c_s_* + 1
**else**: *c_s_* ← 0
**end if**
**if***c_s_* ≥*SI*
**or**
*I* > *MNI*
**then**: output the state *b_i_* as the restored result, end the iteration* Automatic stopping**end if**
**end for**


## 4. Results

Hyperparameters were tested and calibrated so as to provide appropriate settings to the model. SI, MNI and ISR were tested for simulated star images of different amount of noise. Accuracy and running time are the two aspects tested in the experiments. The running time is evaluated by NOI—obviously, more iterations are more time-consuming. Concerning the goal of star image restoration, the accuracy was evaluated by mean squared error (MSE), PSNR, and SSIM, which are defined in Equations (6)–(8) as follow:

(6) $$MSE=\frac{1}{M^{2}}\sum_{i=1}^{M^{2}}([b_{q}^{\prime }{]}_{i}-{\left[b_{q}\right]}_{i}{)}^{2}$$

(7) $$PSNR=10\log_{10}(\frac{MAX^{2}}{MSE})$$

(8) $$SSIM=\frac{\left(2\mu_{b}\mu_{b}\prime +{\left(0.01MAX\right)}^{2})(2\sigma_{bb}\prime +{\left(0.03MAX\right)}^{2}\right)}{\left({\mu_{b}}^{2}+{\mu_{b\prime }}^{2}+{\left(0.01MAX\right)}^{2})({\sigma_{b}}^{2}+{\sigma_{b\prime }}^{2}+{\left(0.03MAX\right)}^{2}\right)}$$

where *MAX* is the possible maximum gray value (which is 255 for 8-bit and 65,535 for 16-bit grayscale image), *b* represents the ground truth and *b′* represents the restore image (also in subscript), *μ* is the average and *σ* is the variance, *σ_bb′_* is the covariance of restore image and ground truth. These three evaluation indicators are calculated for each set of noise images, and then averaged with other sets of noise images with the same type of noise. As described in Section 3.1, there are 1200 sets of noise images with three different levels of noise utilized as the testing data. Thus, the three evaluation indicators are averaged among 400 tests (which is the value range of *q* in Equation (6)) in this study.

**Figure 3:**
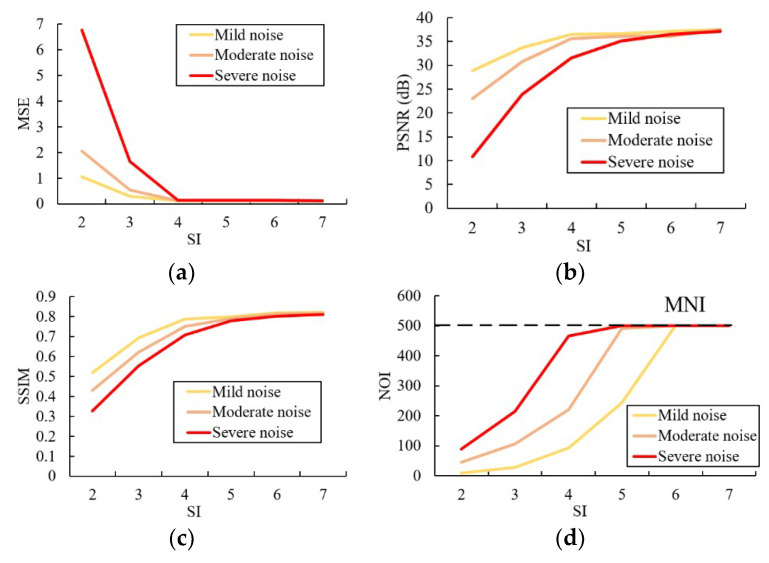
Test results of (**a**) mean square error (MSE); (**b**) peak signal-to-noise ratio (PSNR); (**c**) structure similarity (SSIM); (**d**) number of iterations (NOI) at different settings of stopping index (SI)) for three levels of noise.

The results of experiments on SI and MNI are shown in Figure 3. It turns out that the three evaluation indicators share the similar trend with the change of SI. Specifically, the accuracy of the model improves significantly in a limited range with the increase of SI regardless of the amount of noise in the star images. That means increasing SI in a certain range can improve the quality of restoration results. However, as shown in the same figure, the increase of the SI also leads to more NOI (longer running time). When the SI exceeds certain threshold, the model ends up in infinite loop and has to be stopped by MNI. In that vein, the appropriate SI setting should be large enough to achieve accurate restoration, but small enough to avoid unnecessary iterations.

**Figure 4:**
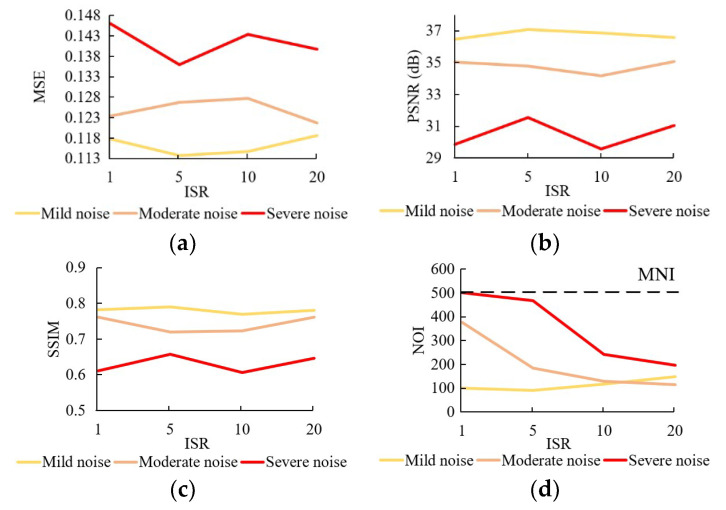
Test results of (**a**) MSE; (**b**) PSNR; (**c**) SSIM; (**d**) NOI at different settings of initial searching radius (ISR)) for three levels of noise.

The results of experiments on the ISR are shown in Figure 4. It turns out that ISR does not have significant influence on the accuracy of restoration results, but affects the running time. Moreover, its influences on running time vary for star images with different amounts of noise: for the star images with mild noise, increasing the ISR does not reduce running time; for the star images with moderate and severe noise, however, the NOI significantly reduced with a larger ISR.

**Figure 5:**
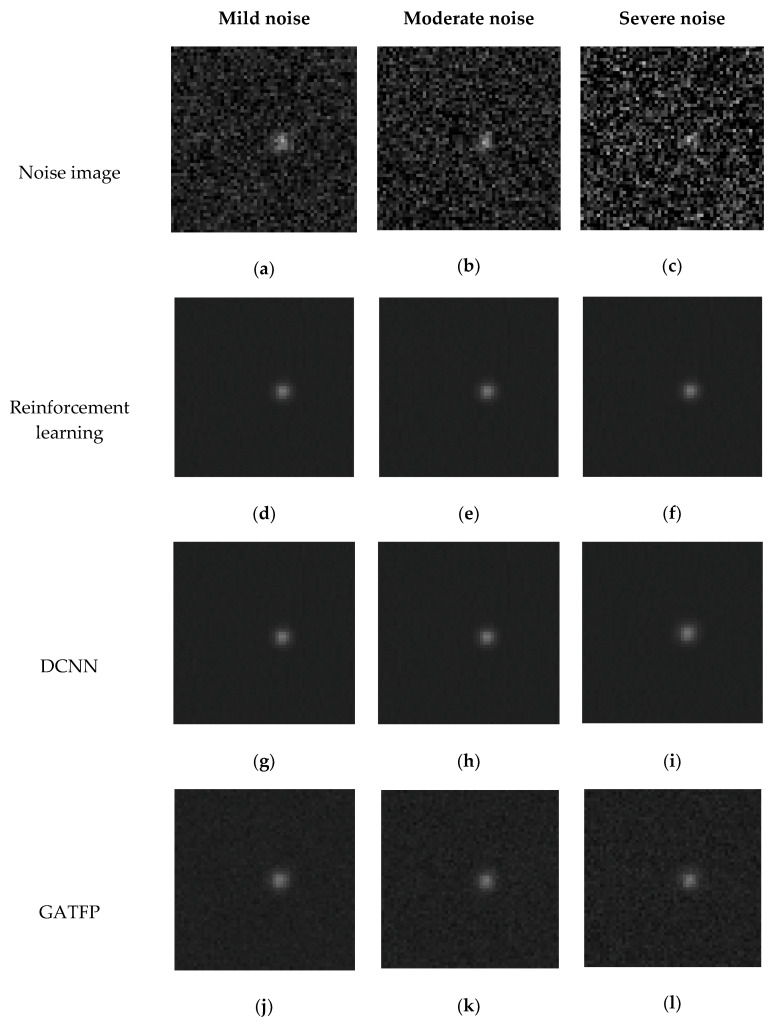
Restoration results on the noise images produced based on the same ground truth (Figure 1): (**a**) noise image with mild noise, (**b**) noise image with moderate noise, (**c**) noise image with severe noise, (**d**) denoising result using reinforcement learning on mild noise, (**e**) denoising result using reinforcement learning on moderate noise, (**f**) denoising result using reinforcement learning on severe noise, (**g**) denoising result using DCNN [45] on mild noise, (**h**) denoising result using DCNN [45] on moderate noise, (**i**) denoising result using DCNN [45] on severe noise, (**j**) denoising result using GATFP [6] on mild noise, (**k**) denoising result using GATFP [6] on moderate noise, (**l**) denoising result using GATFP [6] on severe noise.

The performance of the star image denoising method proposed in this study is compared with GATFP method proposed by Zhang et al. [6] and DCNN algorithm proposed by Liu et al. [45] on the same testing dataset. For the GATFP method, the denoising process is conducted based on the optimized likelihood function given in Equation (3). The implementation of the DCNN model is a setup based on the structure described in Reference [45], which includes 17 convolutional layers and 256 neurons in each layer. Rectified linear unit (ReLU) [54] is applied as the activation function between the layers. The performance of these denoising methods is also evaluated with the three evaluation indicators described above. The arithmetic means of the evaluation indicators for all the testing images in each category of noise are compared in Table 1 for all the three denoising methods. Typical examples of restored images are also shown in Figure 5.

## 5. Discussion

### 5.1. Hyperparameter Settings

#### 5.1.1. SI and MNI

As indicated in the experiment results, SI influences both accuracy and running time. When the SI is set at 2 or 3, the accuracy is relatively low because the decision-making part randomly chooses to stay at the same value. But this possibility quickly decreases as the SI increases to 4. That means the accuracy of the denoising model can be improved by increasing SI in a limited extent: as indicated in Figure 3, the increase of SI does not improve the model accuracy when it is set beyond 5.

NOI also increases with SI. In fact, when SI is larger than 6, the model easily gets into infinite loop and has to be stopped by MNI. This is because in order to stop the iterations without MNI, the decision-making part has to avoid randomly choosing to increase or decrease the gray value for number of times equals to SI after reaching the appropriate restored value—that possibility also decreases with the increase of SI.

It is also notable that the SI has little relation with the amount of noise or searching radius (with adequate iterations). Because the amount of noise decreases with the iterations, and SI does not start to work until the decision-making part reaches to the appropriate restored value. In that vein, 4 or 5 seems to be the appropriate value for SI regardless of the amount of noise in the star image. The MNI setting, however, depends on the amount of noise in the image, since NOI increases with the amount of noise. A short experiment under low SI (for example, 3 or 4) may be necessary to figure out approximately how many iterations the decision-making part needs to form the restored image before formally running the model in order to find the appropriate setting of MNI.

#### 5.1.2. Initial Searching Radius

As indicated in the experiment results, ISR does not affect the accuracy significantly, since it will decrease to 1 in the end. But it affects the NOI. For the images with mild noise, an ISR larger than 1 is unnecessary, and sometimes even causes more iterations, especially when ISR is larger than the noise. For the images with moderate and severe noise, a larger ISR reduces NOI significantly, and therefore, restores the images faster. In practice, however, the quality of observed star image is unknow. In that vein, searching radius is a hyperparameter that needs to be calibrated with actual input star image.

According to discussion of the experiments above, the recommended setting of the hyperparameter for the noise images with different amount of noise using the hyperparameter settings shown in Table 2. This setting is also applied to the training of the reinforcement learning model when comparing its performance with other two methods.

### 5.2. Comparisons with Existing Methods

The algorithm proposed in this article is compared with GATFP [6] and DCNN [45] methods. The performances of the two methods on the dataset used in study generally confirm with those presented in the previous studies. As indicated in Table 1 and Figure 5, these denoising methods are able to suppress the noise in a star image to some extent. The three denoising methods have better performance on the suppression of mild noise than that of moderate noise restoration, which is better than that of extreme noise. This means the images with less noise are naturally easier to be accurately denoised. Although it can hardly be identified from the restored image (Figure 5) by human eyes, the three methods have different performances in term of retrieving the true value of star target. As shown in Table 1, all the three of the evaluation indicators shows that the reinforcement learning method proposed in this study provides more accurate restoration results than that of the DCNN method, which is more accurate than that of GATFP method. That means the algorithm proposed in this article is able to achieve more accurate restoration than the existing star image denoising methods.

The fact that the two machine learning methods achieve better restoration results, especially when the star images are more distorted, is possibly because they overcome the error introduced by the approximation in GAT method. Moreover, the method proposed in this article can perform more accurate restorations than that based on DCNN. It should be noted that the advantage in accuracy of the restoration results is probably caused by the reward function applied in the reinforcement learning algorithm, which is directly linked with the distribution pattern of the noise in the star image. Although the reinforcement learning algorithm proposed in this study is designed more specifically for star image with mixed Poisson–Gaussian noise, it certainly has the potential to be applied to suppress other type of image noise, as long as the corresponding reward function is defined.

In comparing the two machine learning models, the DCNN model [45] includes a series of convolutional layers for image feature extraction and a deep neural network (DNN) for denoising calculation, while the reinforcement learning model presented in this study is an iterative MDP model that is integrated with DNN for action-value regression. The different structures of the two models lead to different training processes. The weights and bias of the neurons are trained in DCNN model, while the optimal denoising process is trained through iterative trials in reinforcement learning model. As a result, the reinforcement learning model is much simpler than the DCNN model in terms of computational complexity. As shown in Table 3, the parameters that need to be trained in the reinforcement learning model is less than one half of those in DCNN model, and the number of computations in training the reinforcement learning model is only about one fourth of that in training DCNN model. This results generally conforms with a comparison between reinforcement learning model and DCNN model by Yu et al. [44].

Moreover, large numbers of noise images for different ground truth are necessary to train DCNN model, while the reinforcement learning model requires much less training dataset: theoretically, the agent can finally propose the restored image even with only one set of noise images with adequate numbers of trials. Thus, the star image denoising model proposed in this study depends on less training dataset, and may be more practical to extract the true value of star target, especially when the observations are limited.

## 6. Conclusions and Future Studies

A novel approach for star image denoising based on reinforcement learning is presented in this study. Compared with the existing methods based on DCNN, this model learns to dynamically search for the appropriate restored image based on reward function derived from mixed Poisson–Gaussian likelihood function. Thus, the mechanism behind our algorithm is more intuitive, and leads to more accurate restoration, than those that are based on DCNN. Compared with MLE methods that try to approximately solve the likelihood function (such as GAT), the same likelihood function is applied as the reward function without approximation. In that way, the likelihood function is solved by the computer through the MDP more accurately.

The limitation of this algorithm is that we didn’t add any kind of convolution layer to the input image. As a result, when the star image is rather huge, the restoration process becomes time-consuming (the accuracy is not affected though). The reason we did not apply CNN in the algorithm is that the reward function derived from the likelihood function only works for the gray value of the star image, but not the image feature as captured by the CNN. An appropriate way to integrate CNN in the algorithm, and thus further improve its efficiency, could be a topic of our study in the future.

It is also a potential topic to extend the application of the reinforcement learning model proposed in this study into the denoising practice of broader types of images, which could have different numbers of color depths. Processing the noise image depth by depth may not be a favorable approach, since it disregards the correlations between the color depths. This problem may also be solved by a properly designed CNN that can extract the features of a noise image.

## Figures and Tables

**Table 1:** Arithmetic means of evaluation indicators for all the testing images with three levels of noise using three different denoising methods.

	Mild Noise			Moderate Noise			Severe Noise		
	MSE	PSNR(dB)	SSIM	MSE	PSNR(dB)	SSIM	MSE	PSNR(dB)	SSIM
Reinforcement learning	0.1147	37.57	0.8215	0.1149	37.10	0.8150	0.1440	31.58	0.7060
DCNN [45]	0.1238	35.43	0.7776	0.1260	35.06	0.7602	0.1587	30.81	0.6816
GATFP [6]	0.1402	31.18	0.7153	0.1638	27.94	0.6384	0.1925	23.05	0.5483

**Table 2:** Hyperparameter settings for the star images of three different amount of noise.

	Mild Noise	Moderate Noise	Severe Noise
Stopping index (SI)	4	4	4
Initial searching radius (ISR)	1	5	10
Maximum number of iterations (MNI)	300	500	500

**Table 3:** Comparison of the computational complexity between the reinforcement learning model proposed in this study and DCNN model [45].

	Reinforcement Learning	DCNN [45]
Parameters (×10^5^)	1.10	2.47
Calculations (×10^8^)	2.26	9.02
